# Making the health system work for over 25 million births annually: drivers of the notable decline in maternal and newborn mortality in India

**DOI:** 10.1136/bmjgh-2022-011411

**Published:** 2024-05-06

**Authors:** Himanshu Bhushan, Usha Ram, Kerry Scott, Andrea Katryn Blanchard, Prakash Kumar, Ritu Agarwal, Reynold Washington, Banadakoppa Manjappa Ramesh, India Exemplars MNH study team, Mitali Raja

**Affiliations:** 1 National Health Systems Resource Centre, New Delhi, India; 2 International Institute for Population Sciences, Mumbai, Maharashtra, India; 3 Institute for Global Public Health, Department of Community Health Sciences, University of Manitoba Rady Faculty of Health Sciences, Winnipeg, Manitoba, Canada; 4 India Health Action Trust, New Delhi, India

**Keywords:** Health policy, Health services research, Health systems, Maternal health, Child health

## Abstract

**Introduction:**

India’s progress in reducing maternal and neonatal mortality since the 1990s was faster than the regional average. We systematically analysed how national health policies, services for maternal and newborn health, and socioeconomic contextual changes, drove these mortality reductions.

**Methods:**

The study’s mixed-methods design integrated quantitative trend analyses of mortality, intervention coverage and equity since the 1990s, using the sample registration system and national surveys, with interpretive understandings from policy documents and 13 key informant interviews.

**Results:**

India’s maternal mortality ratio (MMR) declined from 412 to 103 maternal deaths per 100 000 live births between 1997–1998 and 2017–2019. The neonatal mortality rate (NMR) declined from 46 to 22 per 1000 live births between 1997 and 2019. The average annual rate of mortality reduction increased over time. During this period, coverage of any antenatal care (57%–94%), quality antenatal care (37%–85%) and institutional delivery (34%–90%) increased, as did caesarean section rates among the poorest tertile (2%–9%); these coverage gains occurred primarily in the government (public) sector. The fastest rates for increasing coverage occurred during 2005–2012.

The 2005–2012 National Rural Health Mission (which became the National Health Mission in 2012) catalysed bureaucratic innovations, additional resources, pro-poor commitments and accountability. These efforts occurred alongside smaller family sizes and improvements in macroeconomic growth, mobile and road networks, women’s empowerment, and nutrition. These together reduced high-risk births and improved healthcare access, particularly among the poor.

**Conclusion:**

Rapid reduction in NMR and MMR in India was accompanied by increased coverage of maternal and newborn health interventions. Government programmes strengthened public sector services, thereby expanding the reach of these interventions. Simultaneously, socioeconomic and demographic shifts led to fewer high-risk births. The study’s integrated methodology is relevant for generating comprehensive knowledge to advance universal health coverage.

**WHAT IS ALREADY KNOWN ON THIS TOPIC**

- Many studies in India have analysed specific health and non-health-related factors associated with declines in maternal and neonatal mortality, and particularly proximate interventions, at one time or over short periods. Our study addressed the need to bring together knowledge across multiple levels of drivers, including policy, health programmes, and contextual changes, which have contributed to reduced mortality since the 1990s.

**WHAT THIS STUDY ADDS**

- The study developed an iterative, interdisciplinary methodology to integrate nationally representative quantitative trend data on mortality and coverage with qualitative insights from experts who were involved in developing, implementing, and studying the health policy and systems changes in India since the 1990s. The findings contribute to knowledge on India’s mortality reductions, related improvements in coverage, quality, and equity of MNH interventions, and how these were linked to the government’s policy, administrative and heath systems reforms, alongside contextual changes across this period.

**HOW THIS STUDY MIGHT AFFECT RESEARCH, PRACTICE OR POLICY**

- The study’s positive public health approach can guide future research and action within or beyond India in countries seeking to understand their progress on pressing public health challenges, and build on success for further improvement towards universal health coverage. The study establishes a knowledge platform for cross-learning between states of India, which can also act as a model for other low-income and middle-income countries. The study clearly reflects what, when, and how the health system interacted and worked to reduce mortality.

## Introduction

 Maternal and neonatal mortality declines in India have outpaced global and regional progress, with or without adjustment for economic growth.^1^ According to the sample registration system (SRS), the national maternal mortality ratio (MMR) dropped by two-thirds and the neonatal mortality rate (NMR) halved since 2000. India has contributed greatly to global reductions in maternal and neonatal deaths, as its share of deaths declined from 23% to 12% and 31% to 22%, respectively, between 2000 and 2017.^2 3^

Previous studies in India have analysed specific health and non-health-related factors associated with declines in MMR and NMR,^4^,^8^ and particularly the impact of proximate interventions like antenatal care (ANC) and institutional delivery on NMR.^9^,^14^ However, research is needed to systematically analyse the multilayered factors driving India’s progress. A comprehensive analysis would provide a valuable evidence base to guide India’s national-level efforts to build on its success and for other countries seeking to accelerate progress in maternal and neonatal health (MNH).

The global Exemplars in Maternal and Neonatal Mortality Reduction Study in seven countries is part of Exemplars in Global Health, which includes other subject areas such as child mortality, stunting, community health worker programmes, and vaccine delivery.^15^ As part of this multicountry research, we conducted a mixed-methods study in India to address knowledge gaps and stimulate learning that can inform policy and practice.

India is governed through a federal structure, where the central government sets health policies, priorities, and programmes and provides financing to support these strategies, while the states implement programs and deliver services, while also contributing to their health financing.^16^ Given India’s large and diverse population, a companion paper in this supplement examines two subnational clusters.^17^ This present paper investigates trends in and drivers of India’s maternal and neonatal mortality decline at the national level. The aim of the national analysis was to systematically investigate, document, and compare the contribution of health policies, programmes, and services, as well as changes in coverage, quality, and equity of reproductive, maternal, newborn, and child health (RMNCH) interventions and contextual factors, to the reduction in maternal and neonatal mortality in India over the past three decades.

## Methods

The global Exemplars in Maternal and Newborn Mortality Reduction team was led by the University of Manitoba, Johns Hopkins University, and London School of Hygiene and Tropical Medicine (and guided by an international Technical Working Group of experts). The global team developed criteria to identify countries with faster than expected reductions in NMR and MMR since 2000 relative to their per capita income. In this way, India was identified as having made above-average ‘exemplary’ progress on maternal and newborn survival along with another six countries (Bangladesh, Morocco, Nepal, Senegal, Niger, and Ethiopia); cross-country learnings from all seven countries are presented in Campbell *et al.*^18^

The research in India was jointly implemented by the National Health Systems Resource Centre (NHSRC), the International Institute for Population Sciences, India Health Action Trust, and the University of Manitoba. The NHSRC is a government-funded autonomous body that provides technical and policy support to the Ministry of Health and Family Welfare (MoHFW), Government of India. The MoHFW approved the project and constituted a steering committee and technical working group, which included the leads from the study team and other actors from government, academia, civil society, and the private sector.

### Study design

The global Exemplars MNH study was guided by a conceptual framework (online supplemental figure S1), collaboratively developed to identify and understand the various drivers of change, based on existing interdisciplinary evidence and theory from epidemiology, demography, health policy and systems, and social sciences.^19^ The framework divides the inter-related drivers of maternal and neonatal mortality decline hierarchically into three levels: distal (changes in health policy and systems as well as macrolevel and community-level contextual changes), intermediate (changes in RMNCH+Adolescent (A) programme platforms, health service outputs, and household or individual contextual changes) and proximate (changes in MNH intervention coverage and equity).

The India study team employed a concurrent, multistage mixed-methods design that integrated quantitative data analyses with interpretive understandings from documentary sources and key informant interviews (KIIs) to develop explanations of NMR and MMR reduction.^20^,^22^ Mixed-methods were integrated in two stages. In stage 1, we analysed distributions and trends in maternal and neonatal mortality outcomes, and hypothesised about distribution and changes in coverage (proximate variables) that would have driven the MMR and NMR reduction. Then we analysed distributions and trends in intervention coverage known to be linked to mortality declines, and further hypothesised about programme and service levers, as well as contextual factors, driving intervention coverage and/or mortality reductions. To identify the distal factors of relevance, we qualitatively mapped the timelines of national policies and programmes for providing MNH-related interventions, where and by whom they were to be provided, and the health system changes to support their implementation and service delivery using documentary sources and evidence from among the framework components. We analysed trends in contextual factors that appeared relevant based on existing literature in India. We presented these initial findings in a national stakeholder meeting that convened technical and administrative experts from government, donor agencies, academia, and civil society, and sought input on which policies, health system, and contextual (distal and intermediate) drivers deserve further explanatory analyses in stage 2.

In stage 2, based on the analyses and inputs from stage 1, we analysed available quantitative data on health financing, human resources, and infrastructure, and conducted KIIs to help explain what and how policy, health system, and contextual drivers changed over time in a way that could contribute to intervention coverage and mortality improvements (online supplemental table S1 shows all indicators we analysed among the framework components; more detail on methods below).

We analysed changes in outcomes and drivers during four national policy periods relevant to MNH in India: Child Survival and Safe Motherhood (CSSM) Programme from 1992 to 1997, the Reproductive and Child Health I (RCH I) programme from 1997 to 2005, the RCH II programme, the National Rural Health Mission (NRHM) from 2005 to 2012 and the RMNCH+A programme and National Health Mission (NHM) from 2012 to 2020 (online supplemental figure S2).

### Study setting

India experienced major demographic and socioeconomic changes since 2000 (online supplemental table S2). India’s total population grew by 30%, reaching around 1.38 billion in 2020, with an estimated two-thirds living in rural areas.^23^ Life expectancy increased by 7 years to 69.4 in 2014–2018, nearing the global average of 73.^24 25^ Literacy has improved for men and women. India is an emerging global economy; the gross domestic product has grown by almost 6% annually since the 1990s, though income inequalities have grown.^26 27^

India’s pluralistic health system comprises public providers, informal private providers, and formal private providers across allopathic and indigenous systems of medicine.^16^ About 25% of India’s doctors work solely in the public sector, and around 25% of outpatient and 38% of inpatient healthcare is provided by the public sector.^28^ The government is the predominant provider of immunisation,^29^ ANC,^30^ and institutional delivery.^31^,^34^ In contrast, private providers more often provide curative services.^31^,^34^ Community health workers—primarily Auxiliary Nurse Midwives (ANMs) and Accredited Social Health Activists (ASHAs)—have also been trained by the government to provide preventive, promotive, and some curative MNH services and to connect women and families with formal health services.^35^

### Quantitative data and analysis

An extensive list of indicators was developed, but only indicators with repeated measures at a minimum of two time points since the 1990s could be analysed quantitatively (online supplemental table S2). We reviewed existing nationally representative data sets and analysed the data sets found to be of reasonable quality and comparable over time. We used SRS data^36^ for maternal and neonatal mortality and fertility trends. Pooled data from national household surveys including the National Family Health Survey (NFHS, five rounds,^37^,^41^ and the District Level Household Survey, three rounds^42^,^44^) were used for trends in intervention coverage and equity analyses. Rural Health Statistics,^45^ Health Financing Reports^46^,^50^ and the National Sample Survey Organisation data were used for the number of government health facilities in rural areas, government health expenditure, and the health workforce.

We analysed quantitative trends in NMR, MMR, coverage of key MNH interventions, and contextual changes by computing average annual rates of change (AARC) through regression analysis^51^ for each of the different national policy periods. To measure ANC with contents and intensity-related components, we developed a composite index, referred to as ANCq,^52 53^ which has a 13-point scale. After adaptation to India, our ANCq index consisted of the number of ANC visits, timing of ANC, at least one ANC by skilled provider, blood pressure checked, weight taken, abdomen examined, blood sample collected, urine sample collected, and the number of tetanus toxoid vaccinations given during pregnancy.

For measuring equity in intervention coverage, we used the absolute slope index of inequality (SII) multiplied by 100 (to provide the SII in percentage points). The SII is measured as the absolute difference between the predicted outcome value of those with the highest and lowest scores, after linearly regressing the mid-point of the cumulative proportion of the sample in each population subgroup category (using a score from 0 to 1 from most to least disadvantaged) against the health outcome estimate for each category.^54^ Household wealth tertiles are used instead of quintiles to ensure larger sample sizes particularly in NFHS 1998–1999 and 2005–2006.

We used two analytical approaches to study the impact of fertility changes on maternal and neonatal mortality: (1) the method developed by Jain^8^ using only crude birth rate as fertility measure. This method estimates the maternal/newborn lives saved between two time points in three categories: lives saved by fertility decline, lives saved by newborn health programmes, and lives saved by overlap of both, which Jain refers to as ‘age-parity changes’. However, the method does not use the distribution of births at two time points to estimate the impact of age-parity changes. (2) In order to measure the impact of age-parity changes, we used the bivariate decomposition analysis^55^ with neonatal mortality as the outcome variable, high-risk birth categories as independent variable, and NFHS-3 and 5 as two survey points.

### Qualitative data and analysis

We conducted a comprehensive literature search of published, peer-reviewed articles since 1995. We also conducted a document review of online and archival sources to develop a detailed policy timeline, and to understand health system strategies and implementation processes in the first stage. Then, as mentioned above, a stakeholder meeting was held with 14 experts in June 2021 to present initial results and seek input for identifying key topics for further analysis.

The mixed-methods analyses from stage 1 informed the second stage of qualitative data collection, in which 13 KIIs were conducted by 3 qualitative researchers in the team between July and November 2021 using a semistructured topic guide (online supplemental table S3). We invited 21 experts active since 2000 in MNH policy and implementation from the government, donor organisations, private, civil society, and academia, of which 12 were available and gave oral and written consent. An additional participant was invited to represent the administrative sphere (online supplemental table S4). Interviews on Zoom (averaging 1.5 hours) were audio recorded and transcribed in English. The team coded them in Dedoose software using a codebook based on a priori and emergent topics. Synthesised results were shared with key informants, whose inputs were incorporated.

### Patient and public involvement in research

No patients were involved in this study. Our multi-institutional research team was coordinated by the NHSRC, a Government of India technical support centre. Public involvement in this study occurred through the stakeholder meeting and KIIs.

## Results

### Overall trends in maternal and neonatal mortality

**Figure 1:**
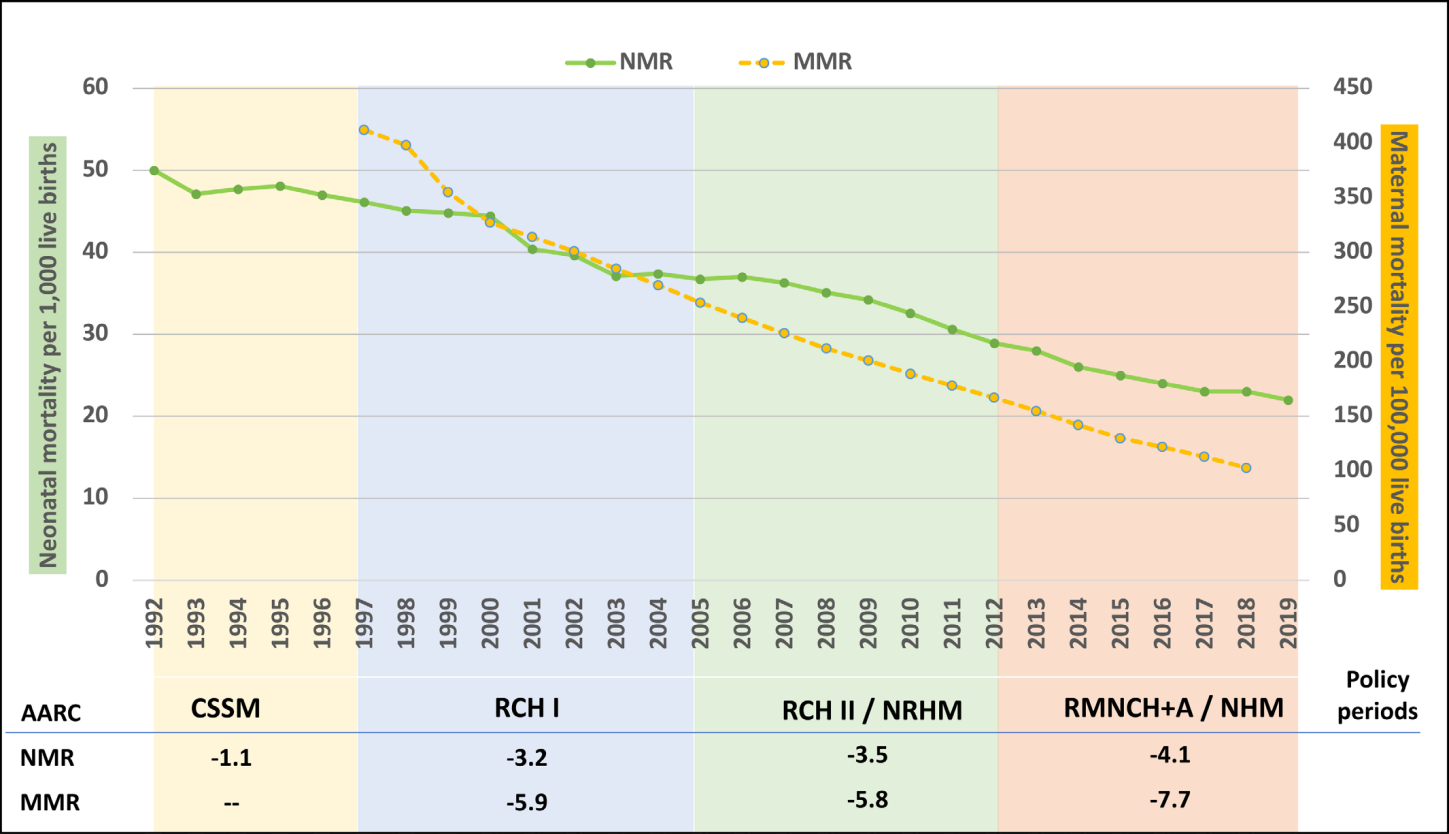
Maternal mortality ratio per 100 000 live births and neonatal mortality rate per 1000 live births, with average annual rate of change (AARC, %) by policy period, India (SRS, 1992–2019). SRS, sample registration system.

Maternal mortality declined from 412 during 1997–1998 to 103 maternal deaths per 100 000 live births during 2017–2019 (figure 1). India’s MMR was already on a declining path before 2000, but the pace of the decline accelerated in this period,^5 56^ with AARC value peaking at −7.7% during 2012–2018. India also achieved a consistent reduction in the NMR, from 50 in the early nineties to 22 per 1000 live births in 2019. The period 2012–2019 recorded the fastest NMR decline (AARC: −4.1%).

### Coverage and equity of key MNH interventions

**Figure 2:**
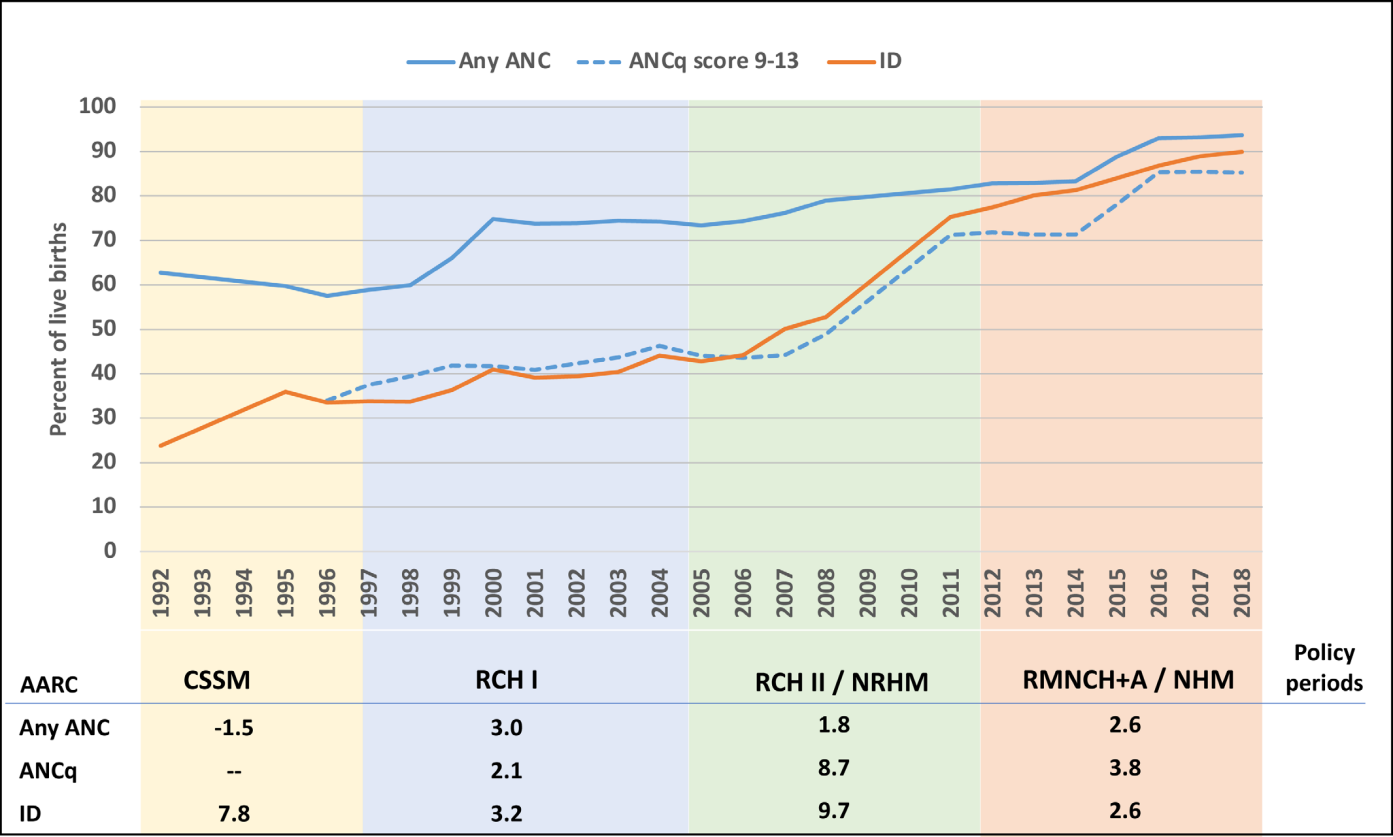
National trends in coverage of antenatal care visits (any ANC), ANC with contents (ANCq) and institutional deliveries (ID, %), with average annual rate of change (AARC, %) by policy period, India (pooled NFHS and DLHS surveys, 1992–2018). DLHS, District Level Household Survey; NFHS, National Family Health Survey.

Increases in coverage of any ANC, ANCq (with contents and intensity), and institutional delivery were gradual (figure 2). The late 90s saw a rapid increase in any ANC, followed by stagnation at around 74% during 2000–2005, following a more gradual increase reaching nearly 94% in 2018. The initially large gap between any ANC and ANCq narrowed during 2005–2012, indicating more ANC visits, early initiation, and better contents of care.^57^ All components of quality improved during the RCH II/NRHM period (online supplemental figure S3).

India’s institutional delivery rate increased slightly since the early nineties, accelerating greatly from 43% in 2005 to 90% in 2019, with the highest AARC during the RCH II/NRHM period. The total number of births in health facilities increased from 11 million in 2005 to 24 million in 2019 (out of around 26 million births in each year).

Coverage of any postnatal care (PNC) for mothers and/or newborns within 48 hours, either in the facility or at home, by a trained professional (nurse, ANM, doctor) or community health worker (ASHA), improved greatly from 38% in 2005–2006 to 83% in 2019–2021 (data not shown).

The public sector share of institutional deliveries increased from about 50% in 1997 to 70% in 2019. The increase in public facility delivery was fastest in the RCH II/NRHM period (online supplemental figure S4). Private facility deliveries increased to 27% of all deliveries but decreased as a share of institutional deliveries from over half to less than a third by 2019. The neonatal mortality among babies delivered in health facilities, both public and private, showed major declines (online supplemental figure S5). Between 2005 and 2020, the proportion of deliveries in the private sector increased only slightly from 20% to 27% and NMR reduced from 37 to 23 while the proportion of deliveries in the public sector increased sharply from 18% to 62% and NMR reduced from 34 to 23 per 1000 live births.

**Figure 3:**
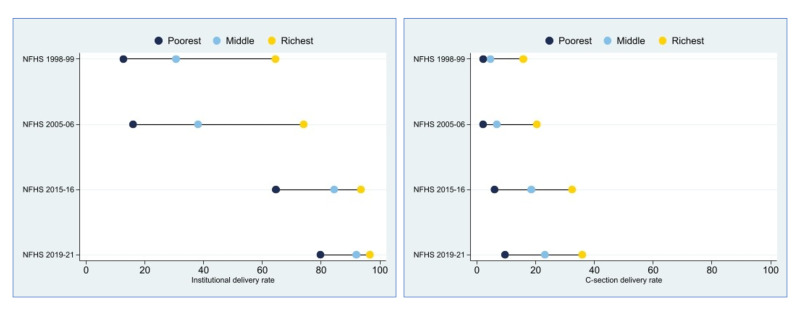
Institutional and C-section deliveries by household wealth tertile, India (NFHS, 1998–2021). NFHS, National Family Health Survey.

The caesarean section (or C-section) rate among all live births increased from just 7% in 1997 to 23% by 2019, paralleling the massive increase in facility deliveries (online supplemental figure S6). While the largest number of C-sections occur in the private sector (often driven by elective procedures), the increases were mainly driven by the public sector where rates started lower. C-sections in the public sector improved faster during the RCH II/NRHM period than previously (AARC: 0.4%) and accelerated a bit in the RMNCH+A/NHM period (AARC: 0.6%). C-section rates among the poorest wealth tertile also increased from 2% in 1998–1999 to 9% in 2019–2021, nearing levels considered to meet population need^58 59^ (figure 3).

Inequalities in coverage of any ANC, ANCq, institutional delivery, and PNC reduced between groups based on urban–rural residence, women’s education, household wealth, and, to a lesser extent, caste/tribe groups (table 1). Inequalities by caste/tribe were already lower in the NFHS 2005–2006. Inequalities were greater in 2005–2006 and reduced more dramatically for ANCq and institutional delivery (though inequalities persist). This is illustrated by the decline in SII from around 60–70 percentage points in 2005–2006 to around 20–30 points in 2019–2021, respectively, between the most and least disadvantaged residence, education, and wealth groups. These reductions in inequality facilitated the massive overall increases in coverage of these interventions across socioeconomic groups (online supplemental table S5). Reduction in inequality in NMR was less dramatic than for coverage. The SII for institutional delivery by household wealth dropped by 60%, while the inequality index for neonatal mortality by household wealth declined only by 8%.

**Table 1:** Absolute inequalities (slope index of inequality, %) in coverage of any ANC, ANCq (ANC with 9±13 contents), ID and PNC within 48 hours of delivery and neonatal mortality per 1000 births (in the 5 years preceding the survey) by selected background characteristics, India (NFHS, 2005–2006 and 2019–2021)

Characteristic	Any ANC		ANCq (score 9–13)		Institutional delivery		Mother or child received PNC within 48 hours		Neonatal mortality	
	2005–2006	2019–2021	2005–2006	2019–2021	2005–2006	2019–2021	2005–2006	2019–2021	2005–2006	2019–2021
Place of residence (rural vs urban)	41.2	5.7	65.4	19.8	64.1	16.8	55.3	12.6	−28.6	−20.3
Woman’s education (no vs some education)	54.6	13.7	77.1	30.5	71.0	29.0	63.6	21.5	−26.8	−21.3
Household wealth tertile (poorest vs richest)	49.2	11.9	77.0	30.1	73.6	29.5	65.7	24.6	−30.4	−28.1
Castes/tribe (scheduled castes/tribe vs others)	11.2	2.3	27.6	5.5	29.8	8.3	21.6	3.3	−13.5	−12.1

All estimates significant at p<0.01.

ANC, antenatal care; ID, institutional deliveries; NFHS, National Family Health Survey; PNC, postnata care.

### Contextual factors

#### Contribution of fertility reduction and birth composition changes

India has experienced steady declines in fertility since the 1970s according to the SRS. The total fertility rate (TFR) declined from 3.3 children per woman in 1997 to replacement level (TFR=2.1) in 2019 (data not shown). While the total number of births in the country remained more or less the same due to population momentum, this rapid fertility decline has resulted in a shift in the age-parity distribution of births. The prevalence of high-risk category births reduced from 46% in 2005–2006 to 29% in 2019–2021 in India, with the greatest reduction in multiple high-risk category births (table 2). In parallel, the neonatal mortality risk among births in the multiple high-risk category reduced from 69 to 45 per 1000 live births. The changes in the composition of births due to fertility reduction contributed 13% of the overall reduction in NMR during this period.

**Table 2:** NMR, birth distribution and results of univariate decomposition by high-risk birth categories, among births in the 5 years preceding the survey, India (NFHS 2005–2006 and 2019–2021)

High-risk birth category	NMR 2005–2006	NMR 2019–2021	Distribution of births		Contribution to NMR reduction	
			2005–2006	2019–2021	Composition	Relative risk
Not in any high-risk category	22.3	18.0	30.1	34.8		
Unavoidable risk category	43.6	26.0	24.2	36.4		
Single high-risk category	39.5	28.9	35.5	23.9	13.1	86.9
Multiple high-risk category	68.7	45.3	10.3	4.9		

NFHS, National Family Health Survey; NMR, neonatal mortality rate.

A birth is classified as high risk if it has one or more of the following characteristics: (1) mother’s age less than 18 years, (2) mother’s age more than 35 years, (3) previous birth interval less than 2 years, and (4) birth order more than 3 years.

The first order births not in any high-risk category were grouped into ‘unavoidable risk category’.^60^

Using the Jain method of decomposition, we estimated the contribution of fertility decline to the reduction in the NMR at 29% (data not shown). The contribution of fertility declines to the absolute number of newborn lives saved between 2000 and 2018 was 46%, including 24% due to fewer births and 22% due to shifts to an age-parity distribution with lower risks of mortality. Fertility decline showed a similar contribution to the MMR reduction (29%) and accounted for around half of the maternal lives saved, considering shifts in age-parity distribution and fewer births.

#### Household living conditions, WaSH, women’s education, empowerment and nutrition

Improved living conditions have been associated with improved birth outcomes in India.^61 62^ Large improvements occurred in household electrification, improved housing materials, use of clean cooking fuel, phone access, and improved drinking water in India (table 3), particularly before 2005 (rates of changes shown in online supplemental table S6). Open defecation has decreased notably from 70% to 20%, with the most rapid improvements occurring more recently.

**Table 3:** Selected indicators of household living conditions, water, sanitation and hygiene, and women’s education, empowerment and nutrition, India (NFHS 1992–1993 to 2019–2021)

	1992–2003	1998–2009	2005–2006	2015–2016	2019–2021
Household living conditions and WaSH (% of households)					
Living in pucca (cement) houses	23.7	32.0	45.9	58.2	60.3
With electricity	50.9	60.1	67.9	88.2	96.5
With landline or mobile	NA	7.4	23.4	90.5	93.4
Using clean cooking fuel	11.6	17.5	25.5	43.8	58.6
With improved source of drinking water	94.0	85.2	87.6	89.9	87.5
With improved sanitation	30.2	36.1	29.1	48.4	69.3
Practicing open defecation	70.0	63.8	55.3	38.9	19.4
With any member having a bank/post office account	NA	NA	40.2	89.4	95.7
Women’s education, empowerment and nutrition					
Median age at first cohabitation, women aged 25–49 years	16.8	17.0	17.4	19.0	18.9
Women age 15–49 who are literate (%)	38.3	46.6	55.1	68.4	71.5
Women age 15–49 with secondary or more education (%)	22.0	29.7	44.7	60.1	65.8
Husband solely decided on wife’s healthcare (%)	NA	39.3	30.1	21.4	16.4
Women age 15–49 having bank account (%)	NA	NA	15.1	52.6	78.4
Prevalence of maternal BMI<18.5 kg/m^2^* (%)	NA	41.6	40.8	25.9	20.3
Prevalence of maternal anaemia* (%)	NA	NA	61.0	57.1	59.9
Prevalence of maternal severe anaemia (%)	NA	NA	1.9	1.0	2.3

* Based on married women with at least one childbirth in last 5 years.

BMI, body mass index; NA, not available; NFHS, National Family Health Survey; WaSH, Water, sanitation and hygiene.

Women’s level of empowerment was also examined using a range of available indicators in the NFHS (table 3). Between 1992–1993 and 2019–2021, women’s age at first cohabitation (after marriage) increased from a median of 17 to 19 years. The proportion of women who were literate also improved from 38% to 72%, and who had secondary education from 22% to 66%, in this time period. The proportion of women whose husbands solely made their health decisions declined from 39% to 16% (1998–1999 to 2019–2021, respectively), while those with bank accounts increased from 15% to 78% since 2005–2006. In terms of women’s nutrition, the proportion of mothers with low body mass index (BMI) more than halved from 42% to 20%. However, anaemia remained at around 60% and severe anaemia at 2%.

### Health policy and systems changes to improve maternal and newborn health

**Figure 4:**
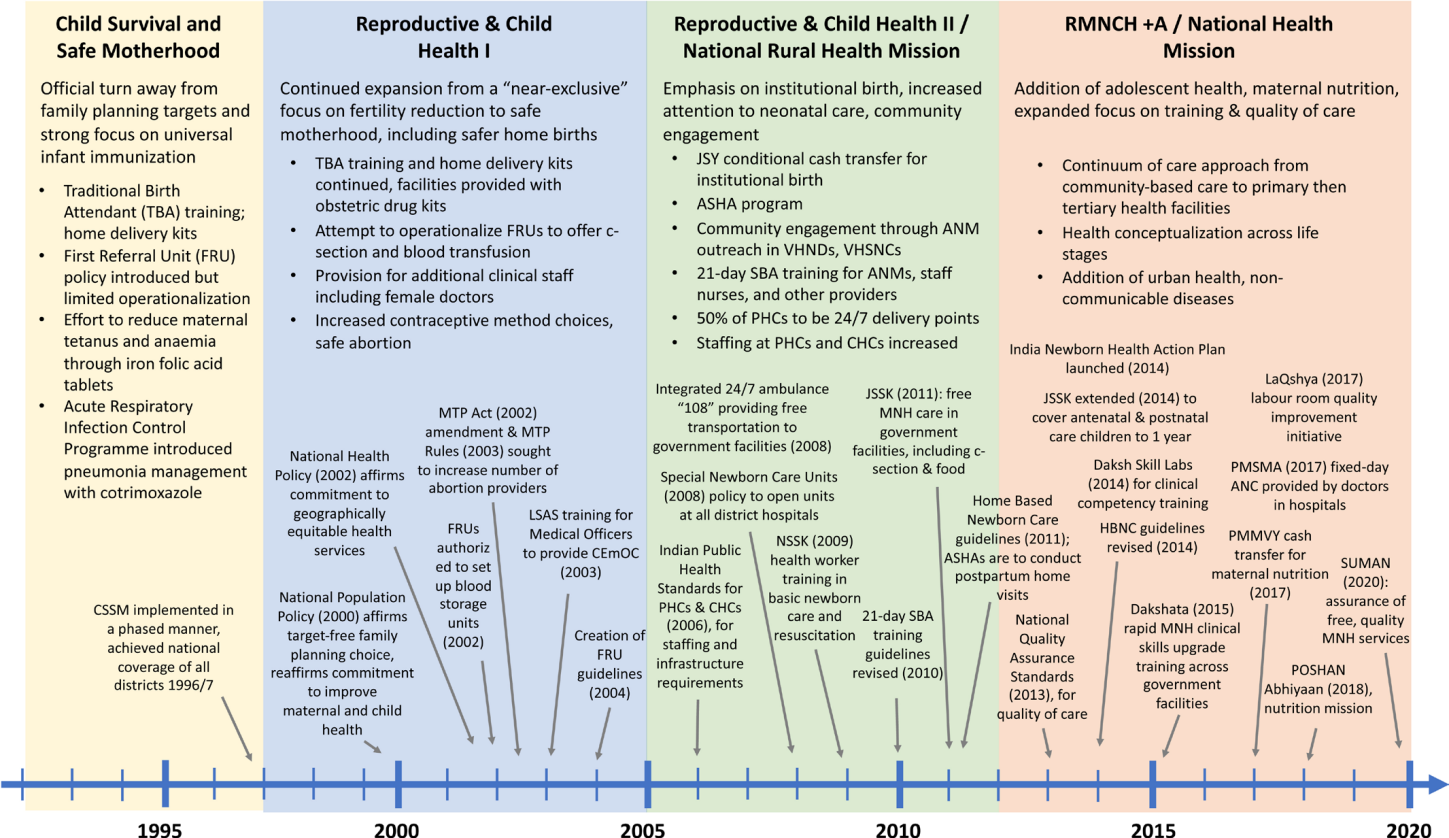
India’s national health policies and programmes related to maternal and newborn health. ANC, antenatal care; ANM, auxiliary nurse midwife; ASHA, accredited social health activist; CHC, community health centre; CSSM, Child Survival and Safe Motherhood; FRU, first referral unit; HBNC, home-based newborn care; JSSK, Janani Shishu Suraksha Karyakaram; JSY, Janani Suraksha Yojana; LaQshya, labour room quality improvement initiative; LSAS, life-saving anesthetic skill; MNH, maternal and neonatal health; MTP, medical termination of pregnancy; NSSK, Navjaat Shishu Suraksha Karyakram; PHC, primary health centre; PMMVY, Pradhan Mantri Matru Vandana Yojana; PMSMA, Pradhan Mantri Surakshit Matritva Abhiyan; POSHAN Abhiyaan, Prime Minister’s Overarching Scheme for Holistic Nutrition (intersectoral mission that includes a focus on early and exclusive breast feeding, nutritional supplementation for adolescent girls, pregnant women and lactating mothers); SBA, skilled birth attendant; SUMAN, Surakshi Matritva Aashwasan; TBA, traditional birth attendant; VHND, village health and nutrition day; VHSNC, village health, sanitation and nutrition committee.

The Government of India’s MoHFW implemented a wide range of health system and policy reforms including administrative changes, particularly in 2005 under NRHM, which the key informants expressed as a ‘game changing moment’ (KI 3, civil society) or ‘tipping point’ for improving MNH (figure 4). The NRHM arose to address a series of needs identified by policy leaders, particularly poor linkages between communities and health services, poor access to core life-saving technical interventions, rigid financial and procurement systems, and centralised administrative power. As an ‘architectural correction’, the NRHM not only strengthened community-based primary care and technical healthcare services but also introduced broad financial and administrative reform of the health system.^63^

[The NRHM was] government-owned, government-funded, taxpayer-supported, with political will at the highest level possible. (KI 10, development partner)

Government spending on maternal and newborn health increased with the launch of the NRHM, almost doubling from Rs.1386 crore (US$187 million) in 2009–2010^64^ to Rs.2652 crore (US$358 million) in 2015–2016^49^ or Rs.519 (US$7.00) to Rs.1069 (US$14.42) per birth^65^ (adjusted to 2020 valuation).^66^ The NRHM (which was extended as the NHM in 2012 to include urban areas) brought renewed urgency to improve public healthcare services, and greater domestic ownership and prioritisation of the maternal and child health agenda.

By greatly augmenting the amount and flexibility of government funds available for health services, decentralising health sector planning to the states, and introducing ‘more rigorous reviews and monitoring’ (KI 12, development partner), the NRHM’s ‘mission mode’ was found to have increased state-level ownership, accountability, and data use for monitoring, while strengthening their adaptation of national policies and programmes for human resources, procurement, logistics, and supplies.

… Prior to that [NRHM], you do not have a concept of even a state-level plan with outcomes and so on. […] Starting from a base where the central government is looking for a plan where you [state governments] come back and commit to a certain level of MMR and so on. You never had that […]

With RCH I [Reproductive and Child Health I, 1997-2005] there was no flexibility at all. The center told you exactly what they should do, how much money you will get and where you’re supposed to spend it. But in the RCH II [synonymous with NRHM] they basically said, “Look, there are wide variations across states, you have unique state level and district level problems, so while certain things may be [fixed], we'll also give you a certain percentage of the money as flexible so you can use that either for innovations or for plugging in gaps. It’s really up to you.” This, I think made a big difference. (KI 5, government administrative & private sector)

These broad policy and administrative changes were found to have catalysed six interrelated trajectories that improved MNH service delivery and related coverage with equity. First, the government offered increasingly comprehensive services across the continuum of care, expanding from a ‘near-exclusive’ focus on fertility reduction to programming for safe motherhood, as well as newborn, child, and adolescent healthcare.

So broadly, if you talk on the CSSM [1992-1997], […] the main focus was population, family planning, and there were of course the components of the maternal health, child health, but it was very limited. […] RCH I [1997-2005] tried to focus the operationalization of the first referral unit by supplying various types of kits. So, it was all vertical supply. So, the top-down approach was there even in RCH I […] It was the game-changer in RCH II and NRHM [2005-2012] when both the things [technical and health system supports] were there. (KI 1, government technical)

Policies also included explicit targeting to reduce geographic disparities between states, districts, and urban and rural populations by increasingly prioritising those with poor intervention coverage or mortality outcomes.

Second, the past two decades saw the introduction and massive scale up of community outreach through the launch of the ASHA community health worker programme and village health and nutrition days, which brought counselling and birth planning into communities.

ASHAs were a game-changer in India’s journey for improved health. (KI 3, civil society)

The other important thing of the Mission is the entire communitisation and the ASHAs. Bringing a field level worker, that was new thing which happened in NHM [NRHM]. […] The big change that we saw in institutional deliveries, I would attribute it also apart from the supply side or the demand on the JSY [Janani Suraksha Yojana incentive scheme], I would also attribute it to the field level worker who actually went around and motivated people to come to the institutions. The home-based newborn care where they could immediately, you know, if the child needed some immediate treatment, they could give a pre-referral dose and then refer the child to the facility. I think that was a game changer that they could, you know, visit homes […] We have a huge force: one million ASHAs. And we invested a lot in their capacity. […] There was constant hand-holding by the supervisors. There is a huge, you know, ASHA mentoring structure which supported them, which involves the civil society also. (KI 13, government administrative)

Since the ASHA programme’s launch in 2005 with the NRHM, India has deployed nearly 1 million trained ASHAs, at a ratio of approximately one ASHA per 979 rural population.^67^ NFHS data show that the proportion of women receiving ANC at a village health nutrition day or other village-level centre increased steadily after 2005 in India overall from 7% in 2001 to 27% in 2018 by year of birth. This was higher than the percentage who received care at public primary health centres (PHCs), community health centres (CHCs) or district hospitals, and now similar to private clinics, which had declined over time (by year of birth). The proportion of newborns who received a home visit from the ASHA within their first month increased from 5% in 2015–2016 to 14% in 2019–2021.

Third, and relatedly, government policies pushed to shift childbirth from homes and traditional birth attendance (TBA) to facilities and skilled birth attendance (SBA). This shift involved ending TBA training and introducing a 21-day SBA training for ANMs and nurses.

Not many people may talk about it or even understand the enormity of it, but I think under the SBA initiative when tasks were shifted and the government allowed and trained ANMs and staff nurses to do some of the skills, I think that was, that would have ended up in saving many, many lives. (KI 3, civil society)

The ‘flagship’ JSY conditional cash transfer scheme played a particularly large role in increasing institutional delivery among the poor and marginalised, as did the expansion of integrated emergency transportation. Although these demand-side programmes shifted community norms from home births to institutional deliveries, when the programme began, government health facilities in some lower per-capita income states ‘did not have any capacity’ to handle this demand (KI 8, civil society).

Fourth, each subsequent national programme since the 1990s intensified efforts to improve the availability of government healthcare facilities in terms of density per population and operating hours, and to increase public-sector health worker availability, particularly through filling vacancies and sanctioning additional personnel. While the number of PHCs and health subcentres per million population remained unchanged nationally starting with the sixth Five Year Plan of 1985–1990, the availability of CHCs increased particularly since the late 1990s and early 2000s (online supplemental figure S7). The AARC in the number of CHCs per million population was the highest during the period 2003–2007, coinciding with the RCH II/NRHM policy period (online supplemental table S6). While there is no comprehensive tracking of the density of public sector health workers, the government sanctioned many additional positions for doctors and nurses/ANMs. In India overall, the density of core health professionals (including allopathic doctors and nurses/midwives) increased from 11 to 17 per 10 000 population during 2004–2005^68^ to 2017–2018.^32^ The density of core health professionals with adequate qualifications nearly doubled during 2011–2012 to 2017–2018.^32^ Efforts to bring sufficient human resources to public facilities are ongoing, including through increased flexibility to recruit specialists.

Health is very much human resource intensive. It cannot be any other way. [It’s not] only doctors, nurses: a whole gamut of allied professionals need to be in place. So, I think this filling the gap of the human resource is a critical factor and whichever states have managed to do that effectively have also been able to deliver and we're seeing the outcomes. (KI 11, academic)

Fifth, India developed guidelines and trainings to expand access to evidence-based life-saving technical maternal and neonatal healthcare, including ‘low-cost, high-impact interventions’ (KI 13, government administrative). This included tetanus toxoid injections, supplementary iron and folic acid tablets, additional anaemia prophylaxis, and high-risk pregnancy screening in the antenatal period. To prevent maternal deaths, more training and equipment was provided at delivery points for active management of third stage of labour, injectable magnesium sulphate for eclampsia, and first dose of antibiotics in cases of delayed postpartum haemorrhage (PPH) or sepsis in the intrapartum period.

70-80% of deaths could be avoided just by a judicious use of managing PPH, oxytocin, misoprostol, having proper antibiotic coverage to prevent sepsis, and giving mag-sulf[ate] for eclampsia; these were all the things nurses could do. […] These were low-hanging fruits, and unnecessarily women were dying, which could be prevented using these simple tools. (KI 12, development partner)

In the neonatal period, antibiotics, vitamin K injection, kangaroo mother care and resuscitation received more attention over time.

Finally, there was a growing emphasis on quality improvement through in-service health worker training and expanded guidelines. This included the 21-day SBA training for ANMs, female health assistants, and staff nurses, and life-saving anaesthetic skills training and emergency obstetric care skills training for general doctors. Essential basic neonatal survival skills training for ASHAs focused on HBNC was introduced in 2011. The RCH II/NRHM period also saw the creation of Indian Public Health Standards for health facilities in 2006, which were updated in 2012. The RMNCH+A/NHM period from 2012 brought Daksh and Dakshata skills training for ANMs, staff nurses, and medical officers. This was accompanied by the 2017 Labour Room Quality Assurance Initiative emphasising patient-centred maternity care, the National Quality Assurance Standards, and Surakshit Matritva Aashwasan, which added a maternal health rights perspective and community participation.

Overall, the key informants expressed diverse opinions on the relative contribution of improvements in the government health system, the private sector, and the social determinants of health. One respondent (KI 8, civil society) considered government programmes to have had minimal impact in India’s lower per-capita income states due to inadequate implementation; this respondent pointed instead to fertility reduction and increased access to life-saving care at private facilities (at high out of pocket costs for families). While implementation challenges were often noted by the other respondents, they also focused on the interrelated improvements to administrative and technical aspects of government-provided healthcare, emphasising that strengthening the underlying health system was the ‘bedrock’ (KI 13, government administrator) on which all other improvements were built. Most respondents considered the private sector to have played a limited role in reducing India’s overall MMR and NMR compared with the ‘massive expansion of public sector’ that has occurred to serve the populations with the highest need.

Private sector has been and will continue to be serving a segment of population which has always been there. And the mortality in that segment of population is miniscule. Because of their overall vulnerabilities and access and their socio-economic status and those kind of things, so these are all confounders; the same kind of people are not going to private, which are going to public. (KI 12, development partner)

All emphasised the importance of fertility reduction, poverty reduction, road connectivity, and improvements to women’s empowerment and education alongside the health-related drivers.

## Discussion

This study sought to understand drivers of India’s above-average progress in reducing maternal and neonatal mortality since the 1990s. Our findings reiterate other analyses^5769^,^75^ showcasing improvements in contact coverage across the continuum of care, particularly in the public sector and among lower socioeconomic and rural groups. AARCs in distal to proximate drivers of NMR and MMR reduction across different levels of the study framework show a sequential pattern of improvement over time (online supplemental table S5). The fastest improvements in contextual drivers (eg, fertility decline, household electricity, and female literacy) occurred during the first policy period (CSSM 1992–1997). Then, the fastest improvement in intermediate drivers (eg, health infrastructure availability) occurred during RCH I. Finally, the fastest improvement in proximate drivers (eg, ANCq, institutional delivery, C-section) and outcomes (NMR and MMR) occurred during the most recent periods. The study results together suggest that the accumulation of sociodemographic contextual changes across India from the late 1990s, and intentional national health systems investments, reforms, and expanded technical services, particularly in the NRHM/NHM period since 2005, accelerated progress in MNH intervention coverage and ultimately mortality reductions among women and newborns in the most recent period.

In examining what has worked and how, we considered the role of the public and private sector, as well as broader socioeconomic development. While enduring challenges in India’s maternal and newborn healthcare services have been well documented—including human resource shortfall in the public sector,^32^ poor quality of care,^76^,^79^ and uneven physical access^80^ and referral pathways to care in rural areas^81^—the results indicate that India made noticeable progress in expanding availability and utilisation of public MNH services. Government expenditure on MNH doubled from 2009 to 2016, while the total number of births in India remained constant. National-level data show that public facility births increased tremendously, particularly for the poor, while private facility birth coverage hardly increased. At the same time that public facilities took on a growing number of deliveries and an increasing portion of all deliveries (reaching 70% by 2018), provision of life-saving emergency services such as C-sections in these facilities increased (as shown in online supplemental figure S6).

The results of this study and others indicate that the NRHM introduced national bureaucratic reforms, additional resources, pro-poor commitments, and accountability through data-driven decision-making, localised monitoring, and community engagement.^63 82^ The Mission introduced JSY conditional cash transfers and the ASHA community health worker cadre, which contributed to rapidly increased demand for ANC and institutional delivery, particularly among the most marginalised.^83^,^87^ Simultaneously, expanded delivery points increased financial and geographical accessibility.^3288^,^91^ Basic and comprehensive emergency obstetric and neonatal care were said to be expanded more recently, though trend data are less available. Our qualitative results and others suggest that this expansion was facilitated by large-scale health worker upskilling with SBA and EmONC training, training CHWs to counsel and support families for family planning, birth preparedness, and home-based newborn care, strengthening digitised systems for procurement and distribution of drugs and equipment, as well as more integrated emergency transportation.^92 93^ Still, ongoing inequities in NMR indicate the interplay of poorer quality of care, use of lower level types of facilities, and problems with emergency referral disproportionately experienced by marginalised groups.^94^

India’s health sector changes occurred within the context of smaller family sizes, increased women’s education, and delayed first pregnancy, which had been shifting already since the 1990s. India also saw overall economic growth, and more recently expanded mobile phone and road networks. In light of existing evidence, it is reasonable to conclude that these contextual changes combined to reduce the number of high risks births, as fertility declined towards replacement level, and to further increase service access, and ultimately, lower mortality risks.^95^,^98^ Public programmes beyond the health sector also simultaneously sought to improve food security and nutrition, though efforts to reduce constant levels of severe anaemia require ongoing attention particularly to address indirect causes of maternal mortality and morbidity.^99^ The rising levels of primary and increasingly secondary education among women may be linked to national policies and programmes, including for universal primary school since 2000, extended into secondary education in 2010 and 2018, and to support equal opportunities and political participation of women, including gender-sensitive policies under NRHM.^100^

The India Exemplars MNH study’s collaborative study approach helped synthesise data to answer complex questions about the drivers of maternal and neonatal mortality reductions. The Global Exemplar study also led to the development of a model that visualises the transition over time from high to low MMR and NMR.^101^ This model identifies areas of focus at the proximate, meso and distal levels for countries and states seeking to move to the next stage in their transition. Further efforts are needed to strengthen data and analyze trends in mortality and related causes, which are currently limited to verbal autopsy, in combination with research on quality of care and other health system gaps that contribute to ongoing NMR inequities.^102 103^ The Exemplars approach can be readily adapted by an array of actors to rigorously generate interdisciplinary knowledge to answer pressing questions and guide future action.^104^

The study faced some limitations. There was no high-quality national-level data measured at more than one time point for many indicators, which left quantitative gaps in our analysis (noted in online supplemental table S1). It was not possible to track stillbirths due to limited samples and trends, and, in particular, widespread under-reporting in NFHS and SRS.^105^ Other key indicators without data were mainly at the intermediate level of our framework, hindering our ability to quantify and link changes in availability of services and contents of care with distal and proximate determinants more directly. National trend data are needed on health facility infrastructure (operation theatres, sick and newborn care unit, etc), staffing/vacancies, drug supply, and evidence-based care provision. While NMR reductions in public facility deliveries can be attributed to a combination of increased provision of life-saving care, changes in case mix (wherein many low risk deliveries that previously occurred at home shifted to public facilities), and improved overall nutrition, data are not available to determine the specific contribution of each of these factors. KIIs may have recall bias around longer-term events and issues, and fewer policy and programme documents were available in the earlier period. Unpacking the persistent NMR inequalities despite huge reductions in coverage inequalities requires more research. The involvement of the MoHFW in approving the study and constituting a steering committee and technical working group could be considered a threat to the independence of the study; however, we consider the Ministry’s involvement to be essential to enhancing the uptake of the results and furthermore did not include the Ministry in analysing or interpreting the data.

## Conclusion

The study results together suggest that the accumulation of socio-demographic contextual changes across India from the late 1990s, and intentional national health systems investments, reforms, and expanded technical services particularly in the NRHM/NHM period since 2005, accelerated progress in MNH intervention coverage and ultimately mortality reductions among women and newborns in the most recent time period. Coverage of public MNH services expanded drastically even for disadvantaged populations, including emergency care to some degree. However, action to address ongoing gaps in service quality and inequities in mortality will be crucial to continue reducing MMR and NMR in India. The mixed-methods study took a comprehensive approach for consolidating knowledge to understand how positive change occurred in MNH, which can motivate future action to advance universal health coverage by ensuring affordable, high-quality healthcare for all.^104^

## Supplementary material

10.1136/bmjgh-2022-011411 online supplemental file 1

## Data Availability

Data are available in a public, open access repository. Data are available on reasonable request.
