# Supplementary material for: Making the health system work for over 25 million births annually: drivers of the notable decline in maternal and newborn mortality in India
Source: BMJ Glob Health. 2024 May 6;9(Suppl 2):e011411. doi: 10.1136/bmjgh-2022-011411 (PMC11085693; doi:10.1136/bmjgh-2022-011411)
Supplement: online supplemental file 1 [file bmjgh-2022-011411supp001.pdf]

Supplementary materials 1: supplementary tables and figures

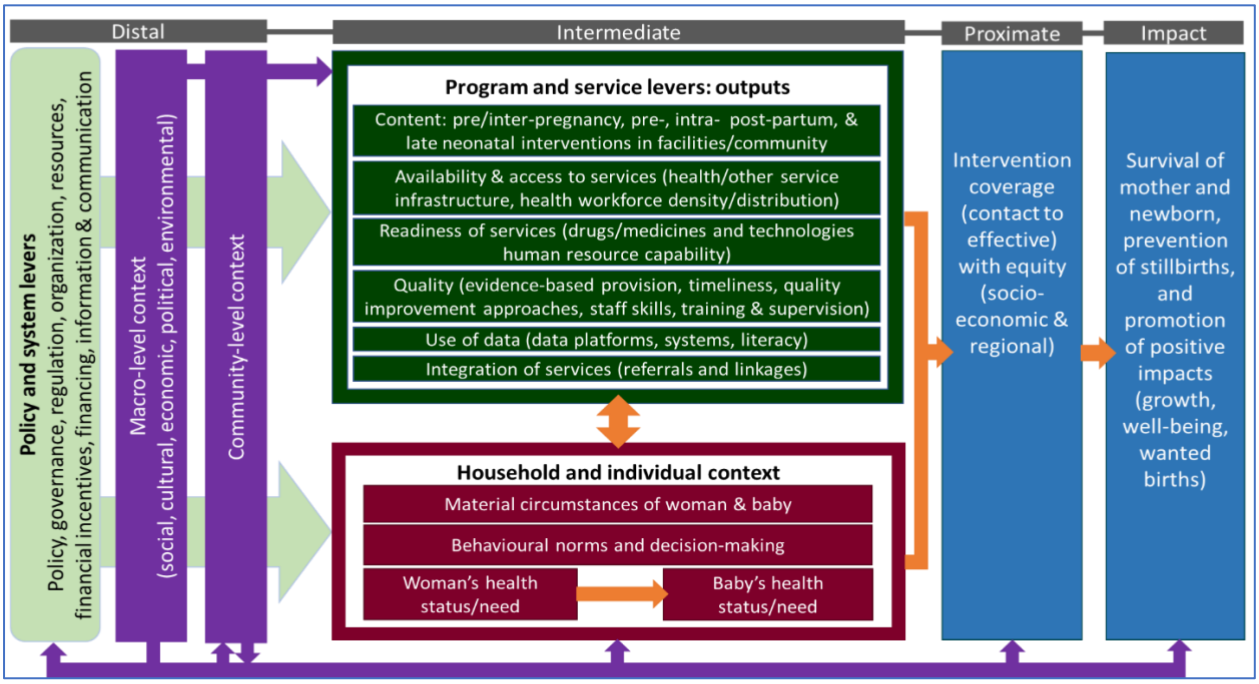

Figure S1: Conceptual framework for the study of drivers of the maternal and neonatal mortality decline, Exemplars MNH study

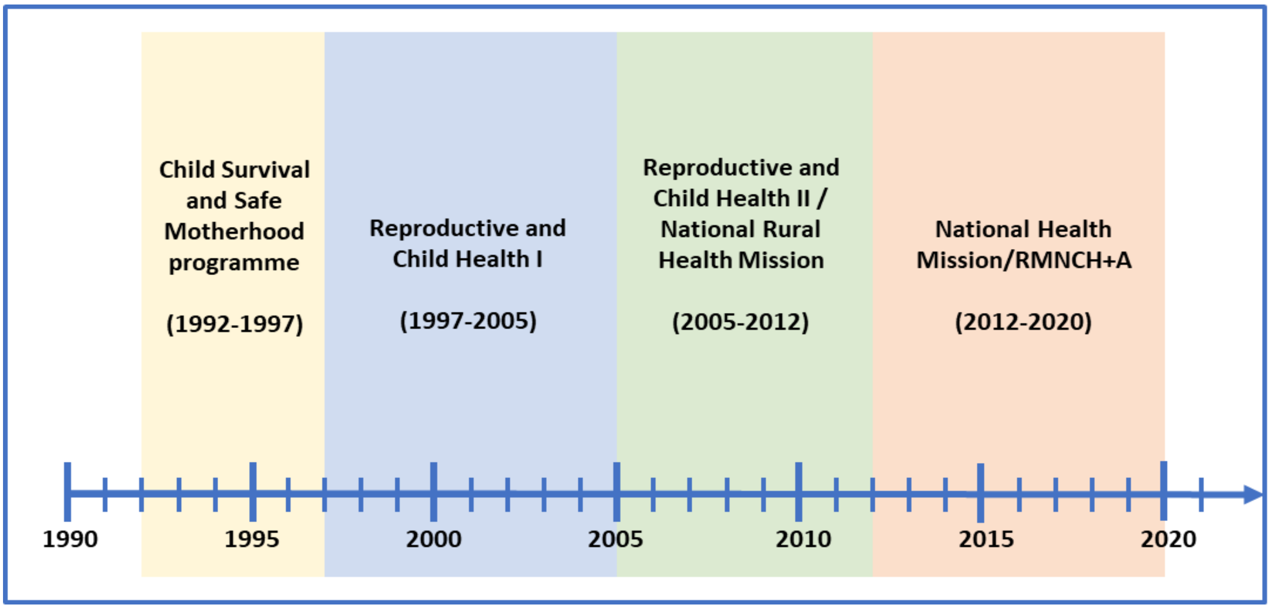

Figure S2: India's health policy periods (1992 – 2020)

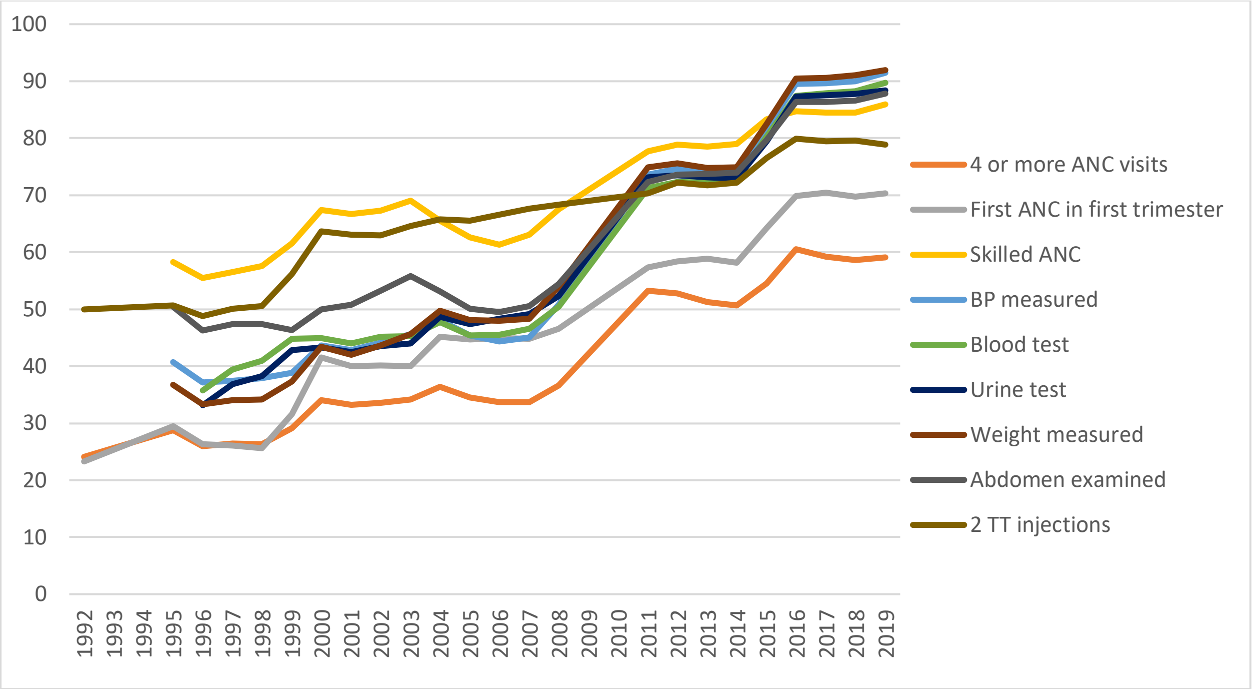

Figure S3: Trends in individual components of ANCq, India (NFHS and DLHS pooled data, 1992-2018)

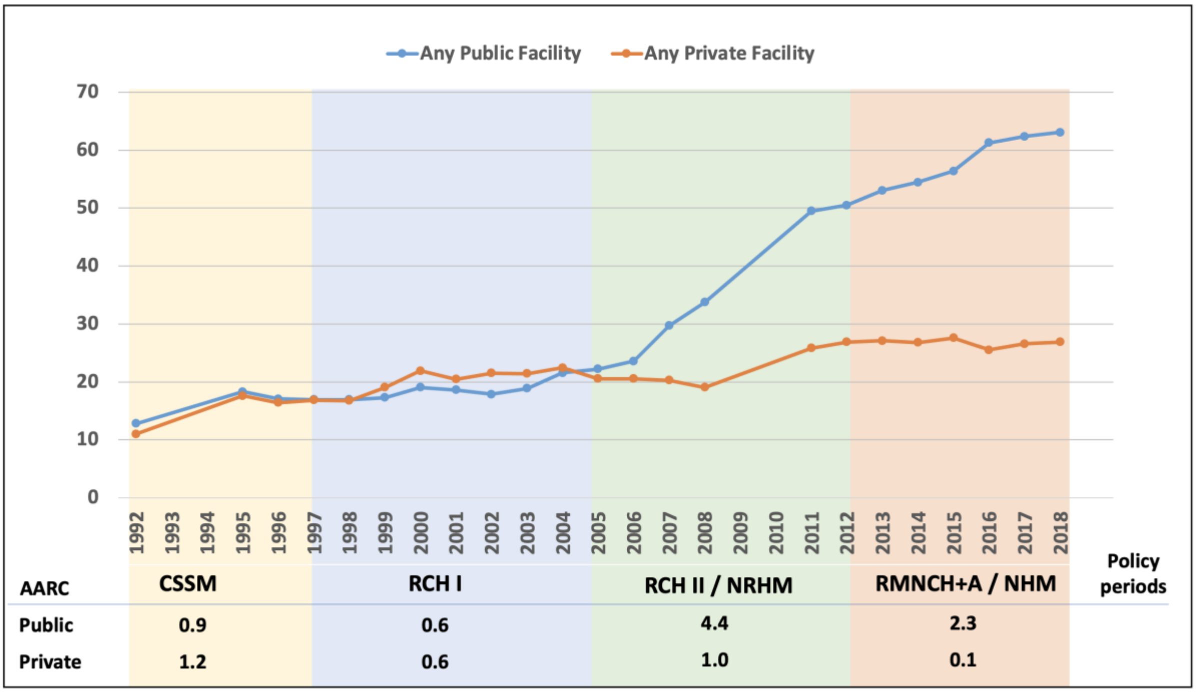

Figure S4: Trends in institutional delivery by facility type, India (NFHS and DLHS pooled data, 1992-2018)

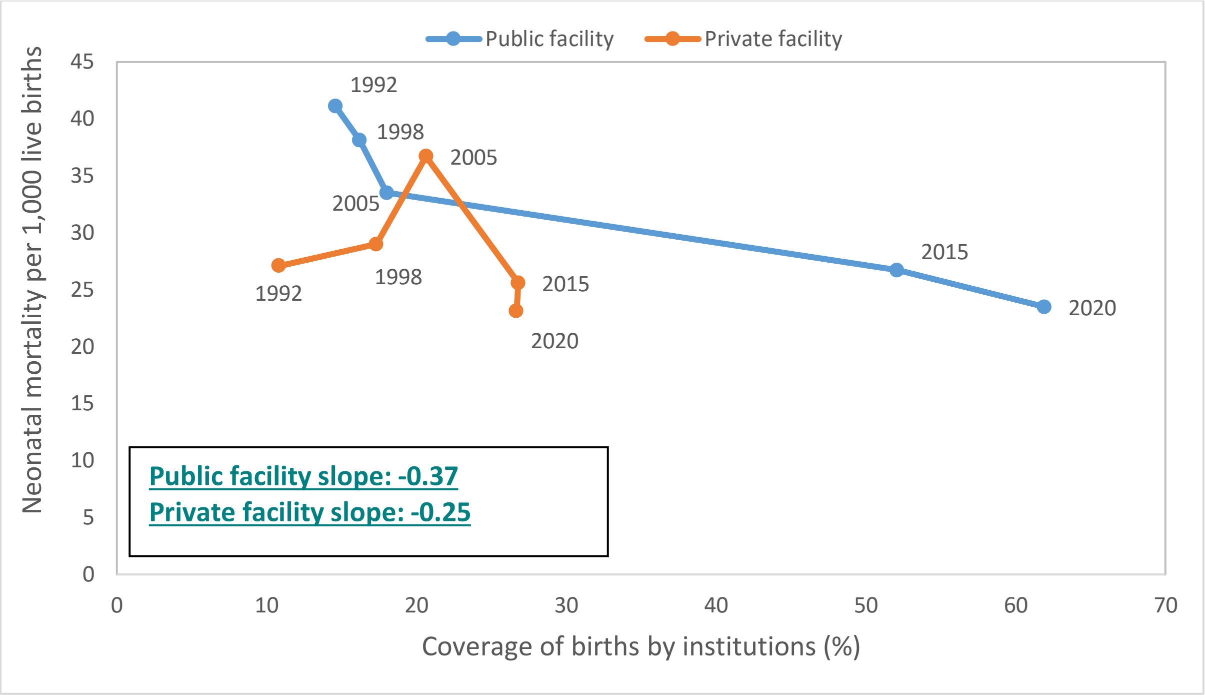

Figure S5: Neonatal mortality per 1,000 live births by institutional delivery coverage, by facility type, India, NFHS, 2005-2020

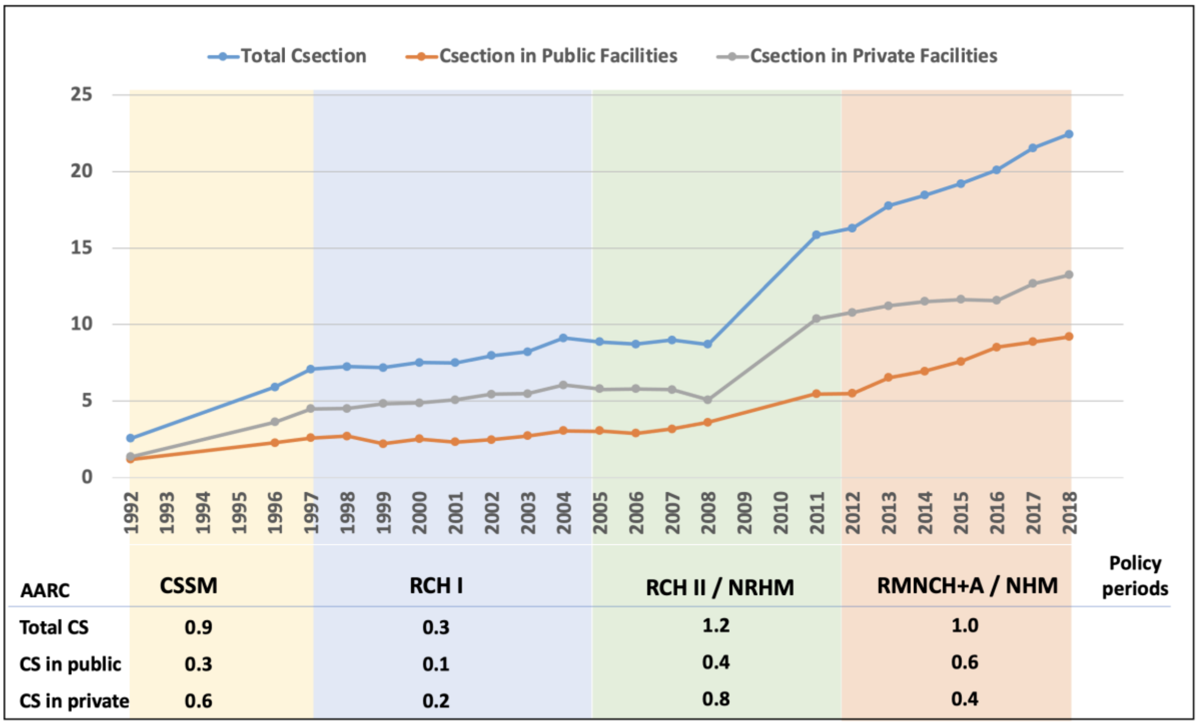

Figure S6: Trends in C-section (CS) rates among all live births by facility type (public and private), India (NFHS and DLHS pooled data, 1992-2018)

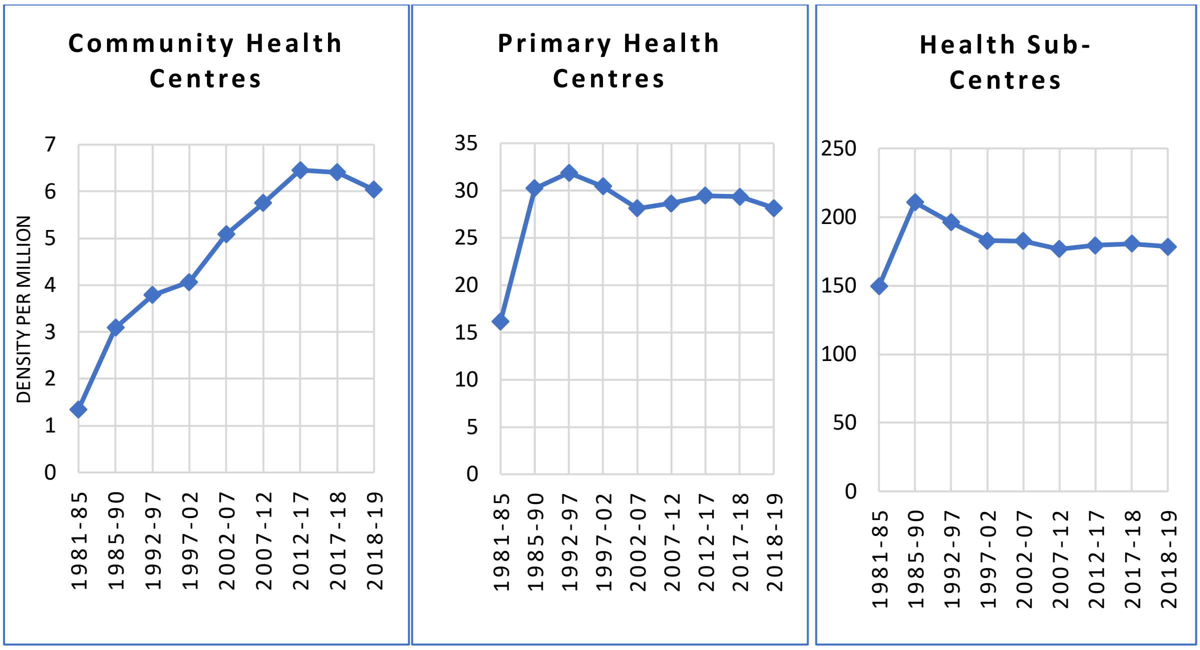

Note: The health facility density is calculated based on population estimated from the RGI projection using the annual exponential growth rate.

Figure S7: Trends in the density of community health centres, primary health centres and health sub-centres per million population in India (Rural Health Statistics 1985-2019)

Table S1. Indicators explored in the multilevel maternal and newborn survival framework

Framework level	Indicators	Data sources and time periods
Impact	• Maternal mortality ratio, average annual rate of change	SRS 2000-18
	• MMR disaggregated by urban/rural, socio-economic status	No national trend data available.
	• Neonatal mortality rate, average annual rate of change	SRS 1971-2018
	• NMR disaggregated by urban/rural, socio-economic status	NFHS-3, NFHS-4 and NFHS-5
	• NMR home births versus institutional births	NFHS, 2005-21
	• NMR public versus private sector institutional births	NFHS, 2005-6 and 2019-21
	• NMR by c-section rate	NFHS, 2005-6 to 2019-21
	• Stillbirths and age-specific NMR[1]	SRS (2000-19), UN-IGME estimates pooling all available data into a global model, NFHS-3 2003 and NHFS-5 2017.

[1] Data on trends in stillbirth was not included because of known underreporting of stillbirths in India. Age-specific NMR trends were analyzed but are not reported in the manuscript because of indistinct trends at the national aggregate level.

Table S1. Indicators explored in the multilevel maternal and newborn survival framework

Framework level	Indicators	Data sources and time periods
	• Causes of maternal death[2]	Special studies related to the SRS, based on verbal autopsy (2001-3 & 2005-6), the Registrar General of India's Medical Certification of Causes of Death (MCCD), HMIS, and subnational research studies. However, a comprehensive assessment of cause-specific trends during 2000-19 is challenging.
	• Causes of neonatal death[2]	Million Death Study (MDS, verbal autopsy 2000 & 2015), the national facility-based MCCD (with more representation of urban health facilities, 2000 & 2019), Global Burden of Disease (2000 & 2019)
Proximate	• Institutional delivery, average annual rate of change	NFHS and DLHS pooled data, 1989-2018
	• Institutional delivery by socio-economic status, urban/rural	NFHS, 1998-2021
	• Institutional delivery by wealth tertile and public/private facility	NFHS, 2005-21
	• Caesarean section rate	NFHS, 1998-2021
	• Caesarean section rate disaggregated by socio-economic status and urban/rural location	NFHS, 1998-2021
	• Caesarean section rate by public versus private facility	NFHS, 2005-21
	• Policies to encourage institutional deliveries	Key informant interviews, stakeholder discussions, document review
	• Antenatal care, average annual rate of change	NFHS and DLHS pooled data, 1989-2018
	• Antenatal care with quality (ANCq), average annual rate of change	NFHS and DLHS pooled data, 1996-2018
	• Share of institutional deliveries at public versus private facilities	NFHS and DLHS pooled data, 1989-2018
	• Share of institutional deliveries at public hospitals, private hospitals and lower level facilities	NFHS, 2005-6 and 2019-21
	• Postnatal care checkup	NFHS, 1998-2021
	• Early essential newborn care, including by facility type (public versus private) for early initiation of breastfeeding	NFHS 2005-6 and 2015-6 for clean cord care, immediate drying, early initiation of breastfeeding, avoidance of pre-lacteal feeds
	• Contraceptive prevalence rate	NFHS, 1998-2021
Intermediate	**Program and service levers**<br>• Content: Interventions for antenatal, intrapartum and post-partum care	Key informant interviews, stakeholder discussions, document review
	• Policy on institutional delivery, community-level primary neonatal care and secondary-level neonatal care	Key informant interviews, stakeholder discussions, document review
	• Availability & access to services:	

[2] Causes of maternal and neonatal deaths were not reported in the manuscript because different data sources and analyses resulted in different findings due to data limitations.

Table S1. Indicators explored in the multilevel maternal and newborn survival framework

Framework level	Indicators	Data sources and time periods
	○ Health infrastructure density	Rural Health Statistics 1985-2019
	○ Proportion women receiving ANC in the community versus private facility, public hospital or PHC/CHC	NFHS, 2005-6, 2015-6 and 2019-21
	○ Contact with a frontline health worker (such as an auxiliary nurse midwife or ASHA) during the third trimester	NFHS, 2005-6, 2015-6 and 2019-21
	○ Communitization of the health system (VHNDs, VHSNCs, ASHA, role of Panchayat raj)	Key informant interviews, stakeholder discussions, document review
	○ Workforce density	Census 2001, NSSO survey, 2004/5, MoHFW statistics, 2005, MoHFW statistics, 2009, NSSO 2011/12, NSSO 2016, NHWA 2018, NSSO 2017/18. Each source has its strength and limitations. A key issue in the surveys and censuses is that a large proportion of respondents defined themselves as employed health workers but lacked the necessary qualifications, especially in the rural areas. Also, the extent to which ANMs are included in survey data is often not clear.
	• Readiness: drugs, medicines and technologies	Key informant interviews, stakeholder discussions, document review. (National level quantitative trend data is not available.)
	• Quality: quality improvement, training and supervision, task sharing	Key informant interviews, stakeholder discussions, document review. (National level quantitative trend data is not available.)
	• Data: use of data platforms and systems	Key informant interviews, stakeholder discussions, document review. (National level quantitative trend data is not available.)
	• Integration: ambulance services and referral systems	Key informant interviews, stakeholder discussions, document review. (National level quantitative trend data is not available.)
	**Household and individual context**<br>• Female empowerment: age at first cohabitation, female literacy, female education, female employment, participation in health-related decision-making, female banking	NFHS, 2005-6 and 2019-21
	• Nutritional status: BMI, anaemia	NFHS, 2005-6 and 2019-21
Distal	**Policy and systems levers**<br>• Policy (periods of reform, political will, targeting of high need states and districts)	Key informant interviews, stakeholder discussions, document review
	• Governance (oversight, monitoring and performance review of states)	Key informant interviews, stakeholder discussions, document review
	• Regulation (creation of guidelines and standards such as the Indian Public Health Standards)	Key informant interviews, stakeholder discussions, document review
	• Financing (per capita expenditure)	

Table S1. Indicators explored in the multilevel maternal and newborn survival framework

Framework level	Indicators	Data sources and time periods
	• Organization (coordination between programs, public and private sectors, development partners)	Key informant interviews, stakeholder discussions, document review
	• Financial incentives (JSY, JSSK)	Key informant interviews, stakeholder discussions, document review
	• Efforts to increase availability of human resources for health, including recruitment and retention, and creating additional posts and cadres	Key informant interviews, stakeholder discussions, document review
	• Out of pocket expenditure (for any health care) per capita and as percent of current health expenditure	World Bank data, 2000-19
	• Out of pocket expenditure (for institutional delivery, by type: c-section or vaginal, and by facility: public or private) and by wealth tertile	NFHS, 2015-16 and 2019-21
	• Health expenditure per capita (US$ 2019 constant)	National Health Accounts 2000-19, GHEx database
	• Total government health expenditure and proportion spent through the NRHM/NHM	NRHM 2005-6 to 2013-4
	**Macro-level context**<br>• Fertility (crude birth rate, absolute number of births per year, total fertility rate, adolescent birth rate), by socio-economic status and urban/rural	Absolute number of births a year derived from population projections and SRS MMR and CBR estimates (2000-18). CBR, TFR, adolescent birth rate from NFHS-2 (1998-9), NHFS-3 (2005-6) and NFHS-5 (2019-21).
	• WASH, telecommunication, electricity, cooking fuel, housing quality (pukka), urban/rural	NFHS, 1998-9 to 2019-21
	**Community level context**<br>• Social movements and civil society pressure to improve maternal and newborn health	Key informant interviews, stakeholder discussions, document review

*Analysis not included in the manuscript due to limitations on the quality of data available

Table S2: Changes in India over the past two decades on selected demographic and socioeconomic indicators, 2000-20

	**2000**	**2020**
**Population**[i]	1.06 billion	1.38 billion
**Life expectancy**[ii]	62.9 years (1998-2002)	69.4 (2014-8)
**Under-five mortality**[iii]	89 per 1000 live births	35 1000 live births (2019)
**Literacy: Male (age 15+)**[iv]	73% (2001)	82% (2018)
**Literacy: Female (age 15+)**[v]	48% (2001)	66% (2018)

**GNI per capita (current USD)**[vi]	$440	$1,900
**GNI per capita, PPP (current international $)**[vii]	$2,070	$6,920
**Gini coefficient**[viii]	74.7	82.3
**Rural population**[ix]	72%	65%

Table S3: Topic guide for in-depth key-informant interviews

The first discussion topic invited respondents to reflect on the overall question:

- From your professional experience and scientific knowledge, how do you think India was able to reduce maternal and neonatal mortality over the last 20 years?

During this initial discussion, respondents were asked to discuss important policies, strategies, and programs and the underlying factors that enabled these policies, strategies, and programs. We provided a wide range of potential probes to expand the discussion on topics relevant to the respondent's area of expertise. For instance, some respondents had more experience on clinical/technical changes (such as treatment protocols and health worker skills) and others on administrative changes (such as management, financing, retention, and recruitment or evaluation); some were maternal health experts, so discussed specific aspects of maternal survival and others focused on neonatology.

The second discussion topic asked respondents to consider differences in high mortality states versus low mortality states. Trigger questions for this topic were:

- What did the low mortality states do to maintain ongoing reductions in maternal mortality? In neonatal mortality?
- What did higher mortality states do to accelerate their reductions in maternal mortality? Sustain their reductions in neonatal mortality?"

The third topic focused on the private sector, asking:

- What role has the private sector played in improving maternal and neonatal survival in India? What is the private sector doing well?
- Over the past 20 years, what has shaped the private sector in terms of its role in providing maternal and neonatal healthcare and the quality of care it provides?
- How has the maternal and neonatal lifesaving capacity of the private sector differed in high versus low mortality clusters?

Fourth, we asked about broader contextual changes, using the discussion question:

- In the past 20-30 years, what are some broader contextual movements, trends, events, or forces that

Table S4: Key informant respondent characteristics

Respondents	N
Gender	
• Male	8
• Female	5
Current affiliation	
• Government: administration	2
• Government: technical	2
• Development partner^	4
• Civil society and academia^	5
Periods active*	
• CSSM (1992-1997)	6
• RCH I (1997-2005)	10
• RCH II/NRHM (2005-2012)	12
• RMNCH+A/NHM (2012-present)	11

^Most development partners and members of civil society and academia had at various points in their careers served as technical advisors to the government through partnerships or involvement in committees. One member of civil society was formerly an employee of the government in a technical role.

* Does not sum to 13 as some respondents were active across multiple policy periods

Table S5: Coverage of any ANC, ANC_q (ANC with contents 9+/13), institutional delivery and PNC (among births in the five years preceding the survey) by selected background characteristics, India (NFHS, 2005-6 and 2019-21)

	**Any ANC**		**ANCq (score of 9-13)**		**Institutional delivery**		**Mother or child received PNC within 48 hours**		**Neonatal mortality**	
**Characteristic**	**2005-6**	**2019-21**	**2005-6**	**2019-21**	**2005-6**	**2019-21**	**2005-6**	**2019-21**	**2005-6**	**2019-21**
**National**	77.2	93.9	45.7	85.7	38.7	88.6	38.3	82.8		
**Place of residence**										
Rural	72.3	93.1	35.5	83.3	28.9	86.7	29.7	81.2	41.8	27.4
Urban	90.7	95.7	73.5	91.9	67.5	93.8	61.8	87.1	28.1	18.0
**Maternal education**										
No Education	62.1	87.3	21.4	70.9	18.4	74.8	20.1	73.1	44.9	33.8
Some Education	90.8	95.5	67.5	89.3	58.9	92.3	54.6	85.2	31.7	22.4
**Household wealth tertile**										
Poorest	65.1	89.3	23.8	74.5	19.3	78.8	20.8	73.9	46.3	32.8
Middle	85.5	95.1	57.3	88.1	48.9	91.0	45.9	84.0	38.8	24.1
Richest	96.7	96.9	85.8	93.7	81.3	96.3	73	89.8	25.5	15.0
**Caste/Tribe group**										
Scheduled Caste/Tribe	73.0	93.1	35.6	83.8	28.1	85.8	30.6	81.7	43.3	28.9
Other castes	79.0	94.2	49.9	86.6	43.3	90.0	41.5	83.4	36.1	23.1

Table S6. Summary of average annual rates of change (AARCs) in different health policy periods for selected indicators at different levels, India, 1992-2020

	Child Survival and Safe Motherhood program (CSSM): 1992-1997	Reproductive and Child Health I (RCH I) program: 1997-2005	Reproductive and Child Health II (RCH II) and National Rural Health Mission (NRHM): 2005-2012	Reproductive, Maternal, Neonatal, Child and Adolescent Health (RMNCH+A) program and National Health Mission (NHM): 2012-2020
**Impact level**				
Maternal mortality ratio	NA	-5.9	-5.8	**-7.7**
Neonatal mortality rate	-1.1	-3.2	-3.5	**-4.1**
Neonatal mortality at days 0-2	-0.5	-0.1	-1.1	**-4.9**
<u>Neonatal mortality by place of delivery</u>				
Home	**-3.0**	0.2	-0.2	**-2.0**
Public hospitals	-1.8	-2.5	-2.2	**-3.4**
Private hospitals	1.0	3.4	**-3.6**	-2.2
Lower level facilities	0.1	0.0	**-4.2**	-3.4
**Proximate level**				
Any ANC	-1.5	**3.0**	1.8	2.6
ANCq	NA	2.1	**8.7**	3.8
Institutional delivery (Total)	7.8	3.2	**9.7**	2.6

Institutional delivery (Public)	0.9	0.6	**4.4**	2.3
Institutional delivery (Private)	**1.2**	0.6	1.0	0.1
C-section (Total)	0.9	0.3	**1.2**	1.0
C-section (Public)	0.3	0.1	0.4	**0.6**
C-section (Private)	0.6	0.1	**0.8**	0.4
**Intermediate level**				
Program and service levers				
Density of HSCs	-1.0	-0.7	-0.7	**-0.1**
Density of PHCs	**0.8**	-1.3	**0.3**	-0.2
Density of CHCs	2.9	**3.0**	2.5	0.2
Density of doctors	NA	NA	**4.4**	0.3
Density of nurses	NA	NA	1.0	**6.2**
Household and individual context				
Median age at 1st cohabitation	0.2	0.3	**0.9**	-0.1
Literacy among women	**3.3**	2.4	2.2	1.1
Secondary+ education among women	5.1	**6.0**	3.0	2.3
Husband+wife decide on health	NA	**11.0**	6.3	2.7
Women having bank account	NA	NA	**13.3**	10.5
Maternal MBI<18.5	NA	-0.3	-4.4	**-5.9**
Maternal anaemia	NA	NA	**-0.7**	1.2
Maternal severe anaemia	NA	NA	**-6.2**	23.1
**Distal level**				
Policy and systems levers				
Health expenditure per capita	NA	8.4	**8.5**	3.8
Macro-level context				
Fertility (TFR)	**-2.9**	-0.9	-2.0	-2.3
Living in pucca (cement) houses	**5.1**	**5.3**	2.4	1.2
Households with electricity	**2.8**	1.8	2.7	2.3
Households with landline or mobile	NA	**17.9**	14.5	0.8
Households using clean cooking fuel	7.1	5.5	5.6	**7.5**
HHs with improved source of drinking water	-1.6	**0.4**	0.3	-0.7
HHs with improved sanitation	3.0	-3.0	5.2	**9.4**
Practicing open defecation	-1.5	-2.0	-3.5	**-16.0**

[i] World Bank. Population total - India [Internet]. World Bank Data. 2021 [cited 2021 Jul 24]. Available from: https://data.worldbank.org/indicator/SP.POP.TOTL?locations=IN.

[ii] Office of the Registrar General and Census Commissioner of India. SRS based abridged life tables 2014-18.Ministry of Home Affairs, Government of India, 2020.

[iii] Registrar General of India. Sample Registration System Statistical Report 2019. New Delhi: Government of India, 2022

[iv] World Bank. Literacy rate, adult male (% of males ages 15 and above) - India. World Bank Data. 2021 [cited 2021 Jul 24]. Available from:https://data.worldbank.org/indicator/SE.ADT.LITR.MA.ZS?locations=IN.

[v] World Bank. Literacy rate, adult female (% of females ages 15 and above) - India. World Bank Data. 2021 [cited 2021 Jul 24]. Available from:https://data.worldbank.org/indicator/SE.ADT.LITR.FE.ZS?locations=IN.

[vi] World Bank. GNI per capita, Atlas method (current US$) – India. [Internet]. World Bank Data. 2021 [cited 2021 Jul 24]. Available from: https://data.worldbank.org/indicator/SP.POP.TOTL?locations=IN.

[vii] World Bank. GNI, PPP (current international $) – India. World Bank Data. 2021 [cited 2021 Jul 24]. Available from: https://data.worldbank.org/indicator/NY.GNP.MKTP.PP.CD?locations=IN.

[viii] Credit Suisse. 2021. The Global wealth report 2021. https://www.credit-suisse.com/about-us/en/reports-research/global-wealth-report.html.

[ix] World Bank. Rural population (% of total population) - India [Internet]. World Bank Data. 2021 [cited 2021 Aug 23]. Available from: https://data.worldbank.org/indicator/SP.RUR.TOTL.ZS?locations=IN.
